# Risk prediction model of dyslipidaemia over a 5-year period based on the Taiwan MJ health check-up longitudinal database

**DOI:** 10.1186/s12944-018-0906-2

**Published:** 2018-11-17

**Authors:** Xinghua Yang, Chaonan Xu, Yunfeng Wang, Chunkeng Cao, Qiushan Tao, Siyan Zhan, Feng Sun

**Affiliations:** 1 School of Public Health, Capital Medical University, 10 Xitoutiao, Youanmen, Beijing, 100069 China; 2 Beijing Municipal Key Laboratory of Clinical Epidemiology, 10 Xitoutiao, Youanmen, Beijing, 100069 China; 3 MJ Health Management Organizations, Taipei, Taiwan; 4 Department of Epidemiology and Biostatistics, School of Public Health, Peking University Health Science Centre, No. 38 Xueyuan Road, Haidian District, Beijing, 100191 China

**Keywords:** Dyslipidaemia, Risk predictive model, MJ longitudinal data

## Abstract

**Objective:**

This study aimed to provide an epidemiological model to evaluate the risk of developing dyslipidaemia within 5 years in the Taiwanese population.

**Methods:**

A cohort of 11,345 subjects aged 35–74 years and was non-dyslipidaemia in the initial year 1996 and followed in 1997–2006 to derive a risk score that could predict the occurrence of dyslipidaemia. Multivariate logistic regression was used to derive the risk functions using the check-up centre of the overall cohort. Rules based on these risk functions were evaluated in the remaining three centres as the testing cohort. We evaluated the predictability of the model using the area under the receiver operating characteristic (ROC) curve (AUC) to confirm its diagnostic property on the testing sample. We also established the degrees of risk based on the cut-off points of these probabilities after transforming them into a normal distribution by log transformation.

**Results:**

The incidence of dyslipidaemia over the 5-year period was 19.1%. The final multivariable logistic regression model includes the following six risk factors: gender, history of diabetes, triglyceride level, HDL-C (high-density lipoprotein cholesterol), LDL-C (low-density lipoprotein cholesterol) and BMI (body mass index). The ROC AUC was 0.709 (95% CI: 0.693–0.725), which could predict the development of dyslipidaemia within 5 years.

**Conclusion:**

This model can help individuals assess the risk of dyslipidaemia and guide group surveillance in the community.

**Electronic supplementary material:**

The online version of this article (10.1186/s12944-018-0906-2) contains supplementary material, which is available to authorized users.

## Introduction

According to the World Health Organization, cardiovascular disease accounts for more than half of all non-communicable diseases and has become the leading cause of death worldwide [1]. Atherosclerosis is the major cause of cardiovascular disease, consistent and convincing evidence supports an association between dyslipidaemia and cardiovascular disease incidence [2]. Dyslipidaemia is defined as an abnormal lipid profile with high triglyceride (TG), total cholesterol (TC) or low-density lipoprotein cholesterol (LDL-C) or low high-density lipoprotein cholesterol (HDL-C). Studies have shown that LDL-C levels are directly associated with an increased risk of coronary artery disease (CAD) [3], whereas higher serum HDL-C levels are a protection factor [4]; however, there is much controversy concerning the proposal that high serum TG levels may be an additional factor for cardiovascular disease [5, 6].

As a result of Westernization of the diet, obesity, and adverse lifestyle changes [7], the prevalence of dyslipidaemia is high and increasing yearly. A report from the American College of Cardiology has indicated that 39% of the global population has elevated cholesterol, and more than one-half of those individuals live in higher income countries [1]. The dyslipidaemia prevalence increased from 18.6% in 2002 [8] to 33.97% in 2010 [9]. Recently, the overall pooled dyslipidaemia prevalence in Chinese adults was estimated to be 41.9% [10]. In Taiwan, the incidence of dyslipidaemia is also high in both adolescents and adults. An epidemiological survey showed that the prevalence of dyslipidaemia significantly increased from 13% in 1996 to 22.3% in 2006 among adolescents [11]. In adults, the prevalence rates of hypercholesterolaemia, hypertriglyceridaemia, an elevated LDL-C level and a low LDL-C level were 53.3% in men and 48.2% in women, 29.3% in men and 13.7% in women, 50.7% in men and 37.9% in women, and 47.4% in men and 53% in women, respectively [12].

Former researchers developed prediction models to calculate a subject’s probability of developing dyslipidaemia [13–15], but their models were not very suitable for the Taiwanese population [16–19]. Therefore, we constructed a risk prediction model of dyslipidaemia among Taiwanese individuals enrolled in the MJ Health Check-up Corporation to evaluate the onset risk of dyslipidaemia in a Taiwanese cohort with periodic check-ups and to provide a reference for individual prevention. The development of this prediction model was based on a cohort with a follow-up of 5 years using clinical information alone or in combination with simple laboratory measures.

## Materials and methods

### Study subjects

The MJ Health Screening Centre comprises 4 health screening centres (Taipei, Taoyuan, Taichung, and Kaohsiung) located around Taiwan. The members involved in the physical examination covered nearly 840,000 people. Their ages range from 18 to 80 years old, and the regional distribution involves 23 cities and counties in Taiwan. Thus, there is a certain representation of the Taiwan population.

The study cohort consisted of 11,345 subjects with the following inclusion criteria: 1) aged 35–74 years; 2) information about the variables was complete; 3) no dyslipidaemia at the beginning of the survey and completed the follow-up in 5 years. The exclusion criteria were as follows: 1) had dyslipidaemia at the beginning of the survey; 2) were using lipid-lowering drugs; 3) were lost in the follow-up and had scant information about the key variables.

### Ethics statement

This study was approved by the Peking University Institutional Review Board, which made the following decision: this study eliminated all identifiable personal information not belonging to studies involving human beings. Thus, we granted waivers of informed consent and ethical review to the study. All or part of the data used in this research were authorized by and received from the MJ Health Research Foundation (Authorization Code: MJHRFB2014003C). Any interpretation or conclusion described in this article does not represent the views of the MJ Health Research Foundation.

### Measurements

The research data were collected in a unified manner that included a questionnaire, physical examination and laboratory testing. The questionnaire included more than 200 indicators, including the demographic data, personal medical history and medication history, family history of cardiovascular disease, physical activity status, smoking status, alcohol consumption level, food type, and symptoms. The physical examination included blood pressure, height, weight, and waist measurements. The laboratory tests included measurements of fasting serum total cholesterol (TC), HDL-C, TG, LDL-C, blood glucose and other routine physical indicators. All the specimens were evaluated at the MJ Central Laboratory, and the biochemical indices were measured in the Hitachi-7150 automatic analyser (Hitachi, Tokyo, Japan) [20].

### Dyslipidaemia diagnostic criteria

Adult dyslipidaemia was defined according to the *Chinese adult dyslipidaemia Prevention Guide*, which was published in 2007 [21] and 2016 [22]. A subject was diagnosed with dyslipidaemia when he/she had one of the following conditions: TC ≥240 mg/dl; TG ≥200 mg/dl; HDL-C < 40 mg/dl in men and < 50 mg/dl in women; LDL-C ≥ 160 mg/dl or non-HDL-C (non-HDL-C = TC - HDL-C) ≥190 mg/dl.

### Statistical analysis

We used the Taipei cohort to develop the model of different dyslipidaemia types. The statistical analysis was completed using SAS 9.1.3. After the medical centre verified the measurement data rigorously, our team performed a comprehensive clean-up of the data and sorted out the follow-up database for analysis. Continuous variables were expressed as the means ± standard deviations, and categorical variables were expressed as percentages.

Based on the logistic regression equation (Eq. 1), we constructed the probability prediction equation (Eq. 2). We used the Hosmer-Lemeshow χ^2^ test to compare the forecasted probability with the actual probability.

1 $$ \mathrm{LogitP}=\ln \left(\frac{p}{1-p}\right)={\beta}_0+\sum \limits_{i=1}^n{\beta}_i{x}_i $$

2 $$ p=\frac{\exp \left({\beta}_0+\sum \limits_{i=1}^n{\beta}_i{x}_i\right)}{1+\exp \left({\beta}_0+\sum \limits_{i=1}^n{\beta}_i{x}_i\right)}=\frac{e^{\mathrm{LogitP}}}{1+{e}^{\mathrm{LogitP}}} $$

To ensure the variables in the preventive model, after adjusting for differences in sex and age, we utilized one-factor logistic regression to determine the role of each variable in dyslipidaemia, used stepwise regression to filter the meaningful variables, and then fit the variables in the prediction model, followed by testing of the predicted probability with a receiver operating characteristic (ROC) curve for diagnosis.

## Results

### Baseline prevalence and 5-year incidence

Table 1 shows the prevalence at baseline of each health screening centre. The prevalence rates of dyslipidaemia in Taipei, Taoyuan, Taichung, and Kaohsiung were 56.5, 56.2, 58.2 and 63.9%, respectively, and the total prevalence was 57.9%.

**Table 1:** Baseline prevalence of dyslipidaemia in the 4 health screening centres

	Taipei	Taoyuan	Taichung	Kaohsiung	Total
Check-up number (*N*)	13,946	4821	3982	4323	27,072
Suffered in dyslipidaemia (*n*)	7877	2708	2317	2764	15,668
Prevalence (%)	56.5	56.2	58.2	63.9	57.9

Note: The prevalence of dyslipidaemia in the table is the crude prevalence and does not standardize by age and sex

Table 2 (Page20) shows the 5-year incidence of dyslipidaemia in the subjects who did not have dyslipidaemia initially. The 5-year cumulative incidence was 19.39%. Those cases of dyslipidaemia included 252 cases with increased LDL-C, 243 cases with increased TG, 629 cases with decreased HDL-C, and 352 cases with increased TC. According to Table 2, most of the “1 item” cases were a HDL-C decrease, and most of the “2–4 item” cases were increases in LDL-C and TC, followed by an increase in TG combined with a decrease in HDL-C.

**Table 2:** Five-year incidence of dyslipidaemia (Page 7)

	Number(all)	Percentage within all (%)	Percentage within dyslipidaemia (%)	Percentage within item (%)
Developed into dyslipidaemia	1170	19.39		
1 item	819	13.58	70.00	
HDL-C	515	8.54	44.02	62.88
TC	135	2.24	11.54	16.48
TG	123	2.04	10.51	15.02
LDL-C	46	0.76	3.93	5.62
2 item	290	4.81	24.79	
LDL-C & TC	167	2.77	14.27	57.59
HDL-C & TG	80	1.33	6.84	27.59
TG & TC	24	0.40	2.05	8.28
LDL-C & HDL-C	18	0.30	1.54	6.21
HDL-C & TC	1	0.02	0.09	0.34
LDL-C & TG	0	0.00	0.00	0.00
3 item	23	0.38	1.97	
LDL-C & TG &TC	10	0.17	0.85	43.48
LDL-C & HDL-C & TC	9	0.15	0.77	39.13
TC & HDL-C & TG	4	0.07	0.34	17.39
LDL-C & HDL-C & TG	0	0.00	0.00	0.00
4 item	2	0.03	0.17	
TC & LDL-C & HDL-C& TG	2	0.03	0.17	
Lipid-lowering medication	36	0.60	3.08	
Remaining normal	4863	80.61		

Note: The incidence of dyslipidaemia in the table is the crude incidence e and does not standardize by age and sex

Table 3 (Page 21) describes the characteristics of the 5-year follow-up of the study population according to the presence of dyslipidaemia.

**Table 3:** Characteristics of dyslipidaemia and non-dyslipidaemia (Page 7)

Characteristic	Training set		Testing set		Total	
	–	+	–	+	–	+
n	4899	1134	4275	1037	19,084	1949
Sex (% man)	43.3	47.7	48.7	52.2	45.5	49.8
Family history of diabetes (%)	20.8	24.0	17.6	19.9	19.3	22.0
Family history of hypertension (%)	34.4	34.1	27.0	29.4	30.9	31.9
Family history of cerebrovascular disease (%)	9.6	10.9	8.7	9.6	9.2	10.3
Family history of cardiovascular disease (%)	12.6	12.9	9.0	10.7	10.9	11.8
Education level lower than junior high school (%)	27.6	33.0	41.7	44.6	34.2	38.6
Marital status						
(% unmarried)	6.4	5.9	2.9	2.5	4.8	4.3
(% married)	85.8	83.6	90.2	89.7	87.8	86.5
(% divorced)	3.2	4.1	1.8	1.6	2.5	2.9
(% widowed)	4.6	6.3	5.2	6.1	4.9	6.2
Exercise						
(% little movement)	39.6	39.6	39.9	40.2	39.7	39.9
(% occasionally movement)	27.9	28.6	26.1	26.5	27.1	27.6
(% exercise regularly)	16.3	16.0	15.3	14.3	15.8	15.2
(% daily exercise)	16.2	15.8	18.8	19.0	17.4	17.3
Overweight and obesity (%)	25.8	38.4	29.5	41.0	27.5	39.7
Current smokers (%)	16.7	19.9	17.8	23.0	17.2	21.3
Current drinkers (%)	22.2	22.6	25.5	27.3	23.7	24.8
Hypertension (%)	14.6	22.4	15.8	18.3	15.2	20.5
High TG level (%)	100.0	0.0	100.0	0.0	100.0	0.0
High TC level (%)	100.0	0.0	100.0	0.0	100.0	0.0
Low HDL-C level (%)	100.0	0.0	100.0	0.0	100.0	0.0
High LDL-C level (%)	100.0	0.0	100.0	0.0	100.0	0.0
High uric acid (%)	13.2	22.9	13.5	19.8	13.4	21.4
Age (years)	45.9	47.58	47.05	47.78	46.44	47.68
BMI (kg/m^2^)	22.31	23.42	22.64	23.60	22.47	23.51
WC (cm)	72.93	74.73	73.54	75.99	73.21	75.29
WHR	0.7800	0.7872	0.7869	0.8017	0.7831	0.7937
Body fat rate (%)	23.60	24.88	24.16	25.65	23.85	25.23
Pulse(times per minute)	72.84	73.35	73.25	74.03	73.03	73.67
FPG (mg/dl)	96.42	98.54	96.84	98.87	96.62	98.70
TC (mg/dl)	192.97	202.37	188.75	199.18	191.0	200.85
TG (mg/dl)	85.66	109.36	85.84	109.22	85.75	109.30
HDL-C (mg/dl)	59.77	56.35	58.71	55.82	59.27	56.10
LDL-C (mg/dl)	116.10	124.17	112.80	121.58	114.56	122.93
SBP (mmHg)	119.0	123.0	119.49	121.18	119.24	122.12
DBP (mmHg)	72.83	74.97	73.02	74.03	72.92	74.51
CRP (mg/dl)	0.2174	0.2728	0.1928	0.2300	0.2085	0.2523
ALP (IU/L)	135.16	143.84	139.16	146.31	137.03	145.02
got (IU/L)	22.33	23.04	23.83	24.22	23.03	23.60
gpt (IU/L)	23.25	25.94	24.59	27.17	23.87	26.53
R-GT (IU/L)	19.48	23.23	21.39	26.94	20.37	25.00
BUN (mg/dl)	14.43	14.67	14.65	14.87	14.53	14.77
CRE (mg/dl)	0.9296	0.9543	0.9634	0.9811	0.9454	0.9671
UA (mg/dl)	5.68	6.12	5.69	6.09	5.69	6.11

Note: “-”means no dyslipidaemia after the 5-year follow-up, “+” means new patients with dyslipidaemia after the 5-year follow-up. Data are expressed as % or means

### Single variable risk analysis (adjusted for age and sex)

According to the risk factors of dyslipidaemia proven in published reports, the value of those factors was explored in the MJ database information. Table 4 (Page 24) lists the relationships between the variables and dyslipidaemia developed using single-variable logistic regression analysis.

**Table 4:** Single-variable logistic regression risk analysis in training set (Page 7)

Variables	Classification value and unit	β	SD_*β*_	*P*-value	RR (95% CI)
Sex	woman				
	man	0.179	0.066	0.007	1.196 (1.051,1.361)
Age		0.018	0.003	0.000	1.018 (1.011,1.025)
Family history	diabetas	0.231	0.079	0.003	1.259 (1.079,1.469)
Education		−0.056	0.031	0.071	0.945 (0.890,1.005)
Drinking	never drinking			0.633	
	current drinker	−0.047	0.086	0.585	0.954 (0.805,1.130)
	quit	0.153	0.217	0.481	1.165 (0.761,1.784)
Exercise	little movement			0.114	
	occasionally movement	0.012	0.082	0.881	1.012 (0.861,1.190)
	exercise regularly	−0.096	0.100	0.337	0.908 (0.747,1.105)
	daily exercise	−0.227	0.104	0.030	0.797 (0.649,0.978)
Body mass index	normal (≤24Kg/m2)			0.000	
	overweight (24-28 K g/m2)	0.502	0.075	0.000	1.653 (1.426,1.916)
	obesity (≥28 K g/m2)	0.627	0.149	0.000	1.871 (1.399,2.504)
BMI	K g/m2	0.111	0.011	0.000	1.117 (1.093,1.142)
WC	cm	0.006	0.003	0.039	1.006 (1.000,1.011)
Weight	Kg	0.035	0.004	0.000	1.035 (1.028,1.043)
FPG	mg/dl	0.005	0.002	0.005	1.005 (1.002,1.009)
SBP	mmHg	0.010	0.002	0.000	1.010 (1.006,1.014)
DBP	mmHg	0.013	0.003	0.000	1.013 (1.007,1.020)
TG	mg/dl	0.018	0.001	0.000	1.018 (1.016,1.020)
TC	mg/dl	0.017	0.002	0.000	1.017 (1.014,1.020)
HDLC	mg/dl	−0.033	0.004	0.000	0.968 (0.960,0.975)
LDLC	mg/dl	0.015	0.002	0.000	1.016 (1.012,1.019)
ALT	IU/L	0.002	0.001	0.033	1.002 (1.000,1.005)
UA	mg/dl	0.204	0.025	0.000	1.226 (1.167,1.289)

Note: the hollow row is the reference group, and RR values are 1.0. The RR values in the table are adjusted for sex and age

### Risk prediction model of dyslipidaemia

The prediction model was named as the MJ Dyslipidaemia Risk Score Model (MJ-DRSM). Table 5 contains information of the model. The prediction model is based on the multivariate logistic regression model. Although differences in the incidence of sex were not obvious after a certain age, all fittings of the prediction models were not divided by gender. The main risk factors in the MJ-DRSM (or the so-called variables) included sex, a family history of diabetes, the HDL-C level, the LDL-C level, the TG level and the BMI. Conversely, exercise, drinking or smoking, and dietary factors were excluded from the model. The included factors were consistent with the aetiology results from a large number of previous studies (both domestic and foreign), met the criteria of risk factors and were confirmed in some large-sample prospective studies.

3 $$ {\displaystyle \begin{array}{l}\mathrm{LogitP}={\beta}_0+\sum \limits_{i=1}^n{\beta}_i{x}_i\\ {}\kern5.5em =-5.2337-0.2290\cdot {x}_1+0.0820\cdot {x}_2-0.00976\cdot {x}_3\\ {}\kern5.5em +0.0134\cdot {x}_4+0.0160\cdot {x}_5+0.0542\cdot {x}_6\end{array}} $$

**Table 5:** Multivariate logistic regression model of all dyslipidaemia

Variables	unit	β	Wald χ^2^ test	*P*-value	RR (95% CI)
Constant term		−5.2337	142.0734	< 0.0001	
sex	1 = male,2 = female	−0.2290	31.0350	< 0.0001	0.633 (0.538–0.743)
diabetes family history	0 = no,1 = yes	0.0820	4.0961	0.0430	1.178 (1.005–1.381)
BMI	K g/m^2^	0.0542	20.3441	<.0001	1.056(1.031–1.081)
TG	mg/dl	0.0160	252.7063	<.0001	1.016 (1.014–1.018)
HDL-C	mg/dl	−0.00976	6.2492	0.0124	0.990 (0.983–0.998)
LDL-C	mg/dl	0.0134	66.9572	< 0.0001	1.014 (1.010–1.017)

According to the parameters listed in Table 5, we can obtain a formula (Eq. 3) to compute LogitP of dyslipidaemia; *x*_1_ - *x*_6_ represent sex, family history of diabetes (0 = no, 1 = yes), HDL-C (mg/dl), LDL-C (mg/dl), TG (mg/dl), and BMI(kg/m^2^), respectively

After calculating the LogitP value of a subject, the probability of an individual developing dyslipidaemia within 5 years can be calculated in accordance with the following formula (Eq. 4):

4 $$ p=\frac{e^{\log \mathrm{itP}}}{1+{e}^{\log \mathrm{itP}}} $$

### Predictive power and cut-off point of the model

**Fig. 1:**
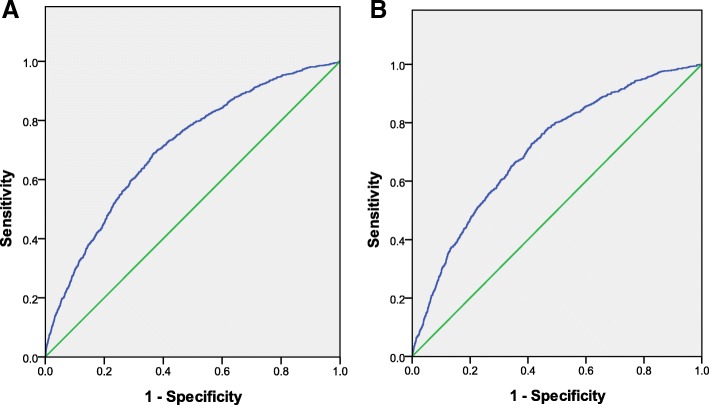
Prediction ability of the MJ-DRSM in Taipei samples and test samples. (**a**) train: AUC=0.707(0.691,0.723) (**b**) test: AUC=0.708(0.691-0.725)

Figure 1a shows the receiver operator characteristic curve (ROC) of MJ-DRSM and the AUC of training set is 0.707 (0.008) (95% CI: 0.691, 0.723). The sensitivity and specificity shows that the best cut-off point is when the predictive probability is *P* = 0.1771, the sensitivity is 68.57%, the specificity is 63.53%, the positive predictive value is 30.33%, and the negative predictive value is 10.27.

### Model validity test

The MJ-DRSM and formula parameters are based on the data of the Taipei medical group, and the model’s validity must be tested in external samples. In this study, we utilized the data of the Taoyuan, Taichung, and Kaohsiung health screening centres to examine the model’s validity. Figure 1b shows the area under the curve AUC = 0.708, indicating that the model has a high degree of curve fitting and good external validity. Thus, the prediction model above can be applied practically to an individual to predict the risk of dyslipidaemia within 5 years.

### Classification of the dyslipidaemia 5-year incidence risk

**Fig. 2:**
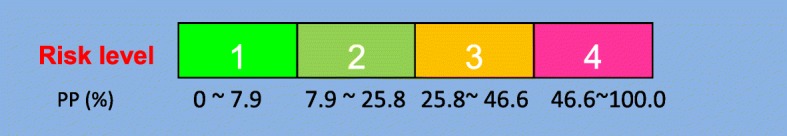
MJ-DRSM prediction probability and risk grade comparison chart

Figure 2 shows the correspondence relationship of the prediction probability and risk level. After transforming the distribution of the 5-year prediction incidence probability into a normal distribution via natural log transformation, we classified the interval of the prediction probability of no dyslipidaemia in the subjects using the mean (M) and standard deviation (SD) and sorted them in ascending order as follows: lower than M-SD was deemed low risk; the range from M-SD to M + SD was deemed general risk; the range from M + SD to M + 2SD was deemed moderate risk; and higher than M + 2SD was deemed high risk.

### Model application

According to the blood test results, the risk of dyslipidaemia within 5 years can be predicted using the following process. The first step is to calculate the LogitP using Eqs. 3 to predict an individual’s risk of dyslipidaemia. Then, the individual’s probability incidence of dyslipidaemia within 5 years can be predicted using eq. 4 to calculate the probability. Finally, Fig. 2 shows the process used to distinguish this person’s risk grade. (More details about the model example are provided in the Additional file 1.)

## Discussion

Estimating the absolute risk required for the prevention or treatment of dyslipidaemia commonly relies on prediction models developed from the experience of prospective cohort studies [23]. Logistic regression analysis was used to examine the associations of some factors with dyslipidaemia in this study. The findings based on the training dataset revealed six common parameters significantly associated with dyslipidaemia (gender, a family history of diabetes, the HDL-C level, the LDL-C level, the TG level and the BMI). Those predictors are consistent with the large number of previous aetiological studies performed worldwide [24–29].

A Japanese study also used logistic regression to evaluate the predictive power of a body shape index (ABSI) for the development of dyslipidaemia; that article focused on the influence of the BMI, WC, and ABSI on the incidence of dyslipidaemia [14]. Another study tried to use plasma free amino acid (PFAA) profiles to predict dyslipidaemia [15]. Their findings suggested that each increase of 1 SD in PFAA index 1 was related to an approximately 34% (12–60%) increased risk of developing dyslipidaemia; for PFAA index 2, the increase was 20% (2–40%). However, the researchers did not illustrate the precision of their model. Our model utilized relatively all-sided information, and its precision was good. Thus, MJ-DRSM was a better choice for predicting the incidence of dyslipidaemia. Recently, some studies had proposed that inflammatory factors such as high-sensitivity C-reactive protein(hs-CRP), Interleukin 6(IL-6) and tumor necrosis factor(TNF) were associated with dyslipidemia, cardiovascular disease and metabolic syndrome [30–33], which was based on the theory that these factors can affect lipid metabolism by promoting the expression of adhesion molecules, the recruitment and activating and gathering the inflammatory cells.

Model sensitivity and specificity are important when testing whether a model can accurately recognize positive and negative outcomes [34]. The ideal model has both high sensitivity and high specificity [35]. The results of the predictive performance showed that the MJ-DRSM model could be used to screen undiagnosed dyslipidaemia patients, because it had good sensitivity (69.9%) and specificity (62.3%). The AUC provides a superior performance index in addition to superior accuracy; therefore, it has often been used to evaluate the predictive accuracy of classifiers [36]. The AUC of a classifier can be defined as the probability of the classifier ranking a randomly chosen positive example higher than a randomly chosen negative example, and higher AUC values can be interpreted as having a higher predictive accuracy [36, 37]. For the MJ-DRSM model, the AUC value was 0.709 (0.693–0.725) in the train cohort and 0.708 (0.691–0.725) in the test cohort. The AUC value of the Japanese study [14] was 0.572 (0.564–0.580). Although the two models were appropriate for different populations, our model contained more variables and had a higher AUC value, which meant that the MJ-DRSM had a better probability of predicting dyslipidaemia accurately. The availability of the predictors is also very important when evaluating whether a model can be feasibly used to identify positive and negative outcomes [13].

To the best of our knowledge, the lipid profile measurement is a standard method to identify and diagnose dyslipidaemia. In this study, we used general epidemiological survey data (gender and a family history of diabetes) with biochemical parameters (HDL-C, LDL-C, TG, and BMI) to develop the MJ-DRSM and distinguish subjects who would be patients with dyslipidaemia after 5 years. To optimize resource use, researchers usually categorize more than just high- and low-risk groups and implement graded intensities of interventions according to the degree of risk [38]. Our data suggest that cut-off points for categorization of risk in the Taiwanese population may be based on the mean and standard deviation (Fig. 2). The high-risk individuals identified will benefit from receiving health education and having the opportunity to engage in healthy lifestyles at an early stage to prevent or delay the onset of dyslipidaemia.

The MJ-DRSM model can accurately calculate the individual probability of dyslipidaemia after 5 years (P1); then, the model puts forward corresponding health suggestions for the subjects and recalculates the 5-year probability after adoption of the proposal (P2). Subjects can compare P1 and P2 to see the advantages of health educational intervention when they follow the advice. Thus, we predict that this model will improve the wiliness of people to change their unhealthy lifestyles according to the health promotive education.

The MJ-DRSM model has a good predictive ability and can directly estimate the 5-year risk of new dyslipidaemia patients in physical examination populations. Additionally, the model can calculate the benefit to the individual after changing the risk factor level to facilitate health education development. Although this study creates a simple scoring tool to predict dyslipidaemia, the study limitations should be noted. First, there were differences in the sociodemographic characteristics between subjects with long-term and rare participation in physical examinations. Second, the model could only be used to predict the 5-year incidence of dyslipidaemia and could not be extrapolated directly to people beyond 35 to 74 years of age. Third, we didn’t measure the plasma hs-CRP, IL-6 and TNF in this study,then we cannot analyze these indexes’ effects on the development of dyslipidemia, we would take those indexes into account in further research. Despite these limitations, the results were based on a large population-based study that combined multiple risk factors, and the prediction model was reliable and effective for the screening of undiagnosed dyslipidaemia patients among the MJ Health screening population.

## Conclusion

The predictability and reliability of our dyslipidaemia risk score model based on the Taiwan MJ Longitudinal Health Check-up Population Database were satisfactory in the testing cohort, with simple and practical predictive variables and risk degree forms. This model can help individuals assess the risk of dyslipidaemia and guide group surveillance in the community.

## Additional file

Additional file 1: Model application. (DOCX 50 kb)

## Abbreviations

ABSI: A body shape index
AUC: The area under ROC
BMI: Body mass index
CAD: Coronary artery disease
HDL-C: High-density lipoprotein cholesterol
hs-CRP: high-sensitivity C-reactive protein
IL-6: Interleukin 6
LDL-C: Low-density lipoprotein cholesterol
M: Mean
MJ-DRSM: MJ Dyslipidaemia Risk Score Model
PFAA: Plasma free amino acid
ROC: The receiver operating characteristic curve
SD: Standard deviation
TC: Total cholesterol
TG: Triglyceride
TNF: Tumor necrosis factor

## References

[1] Laslett LJ, Alagona P, Clark BA, Drozda JP, Saldivar F, Wilson SR, Poe C, Hart M (2012). The worldwide environment of cardiovascular disease: prevalence, diagnosis, therapy, and policy issues: a report from the American College of Cardiology. J Am Coll Cardiol.

[2] Rodrigues AC, Sobrino B, Genvigir FD, Willrich MA, Arazi SS, Dorea EL, Bernik MM, Bertolami M, Faludi AA, Brion MJ (2013). Genetic variants in genes related to lipid metabolism and atherosclerosis, dyslipidaemia and atorvastatin response. Clin Chim Acta.

[3] Grundy SM, Cleeman JI, Merz CN, Brewer HB, Clark LT, Hunninghake DB, Pasternak RC, Smith SC, Stone NJ (2004). Implications of recent clinical trials for the National Cholesterol Education Program Adult Treatment Panel III guidelines. J Am Coll Cardiol.

[4] Gotto AM, Brinton EA (2004). Assessing low levels of high-density lipoprotein cholesterol as a risk factor in coronary heart disease a working group report and update. J Am Coll Cardiol.

[5] Bansal S, Buring JE, Rifai N, Mora S, Sacks FM, Ridker PM (2007). Fasting compared with nonfasting triglycerides and risk of cardiovascular events in women. JAMA.

[6] Nordestgaard BG, Benn M, Schnohr P, Tybjaerg-Hansen A (2007). Nonfasting triglycerides and risk of myocardial infarction, ischemic heart disease, and death in men and women. JAMA.

[7] Wietlisbach V, Paccaud F, Rickenbach M, Gutzwiller F (1997). Trends in cardiovascular risk factors (1984-1993) in a Swiss region: results of three population surveys. Prev Med.

[8] Wu Y, Huxley R, Li L, Anna V, Xie G, Yao C, Woodward M, Li X, Chalmers J, Gao R (2008). Prevalence, awareness, treatment, and control of hypertension in China: data from the China National Nutrition and health survey 2002. Circulation.

[9] Pan L, Yang Z, Wu Y, Yin RX, Liao Y, Wang J, Gao B, Zhang L (2016). The prevalence, awareness, treatment and control of dyslipidaemia among adults in China. Atherosclerosis.

[10] Huang Y, Gao L, Xie X, Tan SC (2014). Epidemiology of dyslipidaemia in Chinese adults: meta-analysis of prevalence, awareness, treatment, and control. Popul Health Metr.

[11] Kuo P, Syu JT, Tzou IL, Chen PY, Su HY, Chu NF (2014). Prevalence and trend of dyslipidaemia from 1996 to 2006 among normal and overweight adolescents in Taiwan. BMJ Open.

[12] Cheng KC, Chen YL, Lai SW (2011). Prevalence of dyslipidaemia in patients receiving health checkups: a hospital-based study. Cholesterol.

[13] Wang CJ, Li YQ, Wang L, Li LL, Guo YR, Zhang LY, Zhang MX, Bie RH (2012). Development and evaluation of a simple and effective prediction approach for identifying those at high risk of dyslipidaemia in rural adult residents. PLoS One.

[14] Fujita M, Sato Y, Nagashima K, Takahashi S, Hata A (2015). Predictive power of a body shape index for development of diabetes, hypertension, and dyslipidaemia in Japanese adults: a retrospective cohort study. PLoS One.

[15] Yamakado M, Nagao K, Imaizumi A, Tani M, Toda A, Tanaka T, Jinzu H, Miyano H, Yamamoto H, Daimon T (2015). Plasma free amino acid profiles predict four-year risk of developing diabetes, metabolic syndrome, Dyslipidaemia, and hypertension in Japanese population. Sci Rep.

[16] Yang J, Li LJ, Wang K, He YC, Sheng YC, Xu L, Huang XH, Guo F, Zheng QS (2011). Race differences: modeling the pharmacodynamics of rosuvastatin in Western and Asian hypercholesterolemia patients. Acta Pharmacol Sin.

[17] Frank AT, Zhao B, Jose PO, Azar KM, Fortmann SP, Palaniappan LP (2014). Racial/ethnic differences in dyslipidaemia patterns. Circulation.

[18] Sumner AE (2009). Ethnic differences in triglyceride levels and high-density lipoprotein lead to underdiagnosis of the metabolic syndrome in black children and adults. J Pediatr.

[19] Lin SX, Carnethon M, Szklo M, Bertoni A (2011). Racial/ethnic differences in the association of triglycerides with other metabolic syndrome components: the multi-ethnic study of atherosclerosis. Metab Syndr Relat Disord.

[20] Wen CP, Cheng TY, Tsai MK, Chang YC, Chan HT, Tsai SP, Chiang PH, Hsu CC, Sung PK, Hsu YH (2008). All-cause mortality attributable to chronic kidney disease: a prospective cohort study based on 462 293 adults in Taiwan. Lancet (London, England).

[21] Chinese guidelines on prevention and treatment of dyslipidaemia in adults (2007). Chinese Circ J.

[22] Junren Chu RG, Zhao S, Lu G, Dong Z, Li J. Guidelines for prevention and treatment of dyslipidaemia in Chinese adults (revised in 2016). Chinese Circ J. 2016;(10):937–53.

[23] Sun F, Tao Q, Zhan S (2009). An accurate risk score for estimation 5-year risk of type 2 diabetes based on a health screening population in Taiwan. Diabetes Res Clin Pract.

[24] Cifkova R, Krajcoviechova A (2015). Dyslipidaemia and cardiovascular disease in women. Curr Cardiol Rep.

[25] Bayram F, Kocer D, Gundogan K, Kaya A, Demir O, Coskun R, Sabuncu T, Karaman A, Cesur M, Rizzo M (2014). Prevalence of dyslipidaemia and associated risk factors in Turkish adults. J Clin Lipidol.

[26] Nuotio J, Oikonen M, Magnussen CG, Viikari JS, Hutri-Kahonen N, Jula A, Thomson R, Sabin MA, Daniels SR, Raitakari OT (2015). Adult dyslipidaemia prediction is improved by repeated measurements in childhood and young adulthood. The cardiovascular risk in young Finns study. Atherosclerosis.

[27] Deedwania PC, Pedersen TR, DeMicco DA, Breazna A, Betteridge DJ, Hitman GA, Durrington P, Neil A (2016). Differing predictive relationships between baseline LDL-C, systolic blood pressure, and cardiovascular outcomes. Int J Cardiol.

[28] Fahed AC, Habib RH, Nemer GM, Azar ST, Andary RR, Arabi MT, Moubarak EM, Bitar FF, Haddad FF (2014). Low-density lipoprotein levels and not mutation status predict intima-media thickness in familial hypercholesterolemia. Ann Vasc Surg.

[29] Shen Z, Munker S, Wang C, Xu L, Ye H, Chen H, Xu G, Zhang H, Chen L, Yu C (2014). Association between alcohol intake, overweight, and serum lipid levels and the risk analysis associated with the development of dyslipidaemia. J Clin Lipidol.

[30] Clearfield MB (2005). C-reactive protein: a new risk assessment tool for cardiovascular disease. J Am Osteopath Assoc.

[31] Koutouzis M, Rallidis LS, Peros G, Nomikos A, Tzavara V, Barbatis C, Andrikopoulos V, Vassiliou J, Kyriakides ZS (2009). Serum interleukin-6 is elevated in symptomatic carotid bifurcation disease. Acta Neurol Scand.

[32] Thomas NE, Rowe DA, Murtagh EM, Stephens JW, Williams R (2018). Associations between metabolic syndrome components and markers of inflammation in welsh school children. Eur J Pediatr.

[33] Imai Y, Dobrian AD, Weaver JR, Butcher MJ, Cole BK, Galkina EV, Morris MA, Taylor-Fishwick DA, Nadler JL (2013). Interaction between cytokines and inflammatory cells in islet dysfunction, insulin resistance and vascular disease. Diabetes Obes Metab.

[34] Ho WH, Lee KT, Chen HY, Ho TW, Chiu HC (2012). Disease-free survival after hepatic resection in hepatocellular carcinoma patients: a prediction approach using artificial neural network. PLoS One.

[35] Walker HKH, Hurst JW (1990). Clinical methods: the history, physical, and laboratory examinations.

[36] Linden A (2006). Measuring diagnostic and predictive accuracy in disease management: an introduction to receiver operating characteristic (ROC) analysis. J Eval Clin Pract.

[37] Ke WS, Hwang Y, Lin E (2010). Pharmacogenomics of drug efficacy in the interferon treatment of chronic hepatitis C using classification algorithms. Advances and applications in bioinformatics and chemistry : AABC.

[38] Tunstall-Pedoe H, Woodward M (2006). By neglecting deprivation, cardiovascular risk scoring will exacerbate social gradients in disease. Heart.

