# Supplementary material for: Risk prediction model of dyslipidaemia over a 5-year period based on the Taiwan MJ health check-up longitudinal database
Source: Lipids Health Dis. 2018 Nov 17;17:259. doi: 10.1186/s12944-018-0906-2 (PMC6240269; doi:10.1186/s12944-018-0906-2)
Supplement: Supplementary file 1 — Model application. (DOCX 50 kb) [file 12944_2018_906_MOESM1_ESM.docx]

Model application

Example: a female membership come to take the physical examination, we known that she has family history of diabetes, her BMI=27.2kg/m^2^ and her lipids level in blood are HDL-C: 52mg/dl, LDL-C: 150mg/dl, TG: 180mg/dl.

We predict her risk of dyslipidaemia in 5 years.

First step is to calculate the LogitP by into the equation 3, 4, 5, 6, to predict individual’s risk for all type of dyslipidaemia, dyslipidaemia of increased in LDL-C, dyslipidaemia of increased in TG or decreased in HDL-C, dyslipidaemia of increased in LDL-C and TG or decreased in HDL-C, dyslipidaemia of increased in non-HDL-C, respectively. Then, predict the individual’s probability incidence of dyslipidaemia within 5 years by into equation 8, which is for calculating the probability. Finally, according to the figure 1 to distinguish this person’s risk grade.

Here, we use equation 3 to calculate this person’s probability risk of dyslipidaemia within 5 years as an example:

The formula (Equation 3) to compute LogitP for all types of dyslipidaemia, and *x*_1_ ~ *x*_6_ represent sex, family history of diabetes (0=no, 1=yes), HDL-C (mg/dl), LDL-C (mg/dl), TG (mg/dl), and BMI, respectively.

$\begin{matrix} \text{LogitP}=\beta_{0}+\sum_{i=1}^{n} \beta_{i}x_{i} \\ =-5.2337-0.2290\cdot x_{1}+0.0820\cdot x_{2}-0.00976\cdot x_{3} \\ +0.0134\cdot x_{4}+0.0160\cdot x_{5}+0.0542\cdot x_{6} \end{matrix}$ Equation 3

So, her logitP of dyslipidaemia is 0.22672, then use this logitP to calculate the P value via Equation 8.

$p=\frac{e^{\text{logitP}}}{1+e^{\text{logitP}}}$ Equation 8

Her probability risk within 5 year of dyslipidaemia is 55.64%.

1

0 ~ 7.9

2

3

4

7.9 ~ 25.8

25.8~ 46.6

46.6~100.0

**Risk level**

PP (%)

Figure 1 MJ-DRSM prediction probability and risk grade comparison chart

It can be seen in the figure 1 above that she is in risk level 4.

Then we can offer an individualized intervention and evaluate it. As this person’s risk rating is the highest level (level 4), in order to reduce she’s risk, we propose the following two intervention strategies, and quantitative assess the expected effect of each strategy.

Intervention strategy one: lose weight. The goal is to reduce the BMI to 24.0, while paying attention to the adjustment of dietary structure, the fat intake limit, the target is HDL-C 55 mg/dl, LDL-C 120 mg/dl, TG 150 mg/dl, if she can successfully reach the target, then her 5 years probability incidence of dyslipidemia will be 29.78%. Her risk level will be in level3.

We can see that if the subjects participate in physical exercise and other weight-loss activities to reduce the BMI to the normal upper limit 24.0, mean while adjusting the diet to lipid measurement decreased, the incidence of dyslipidemia within 5 years will be reduced to 29.78%, the probability risk of reducing 25.86 %. But only decline one level of the risk level, there is still the need for further intervention.

Intervention strategy two: keep going the losing weight diet and exercise to maintain her BMI to 22.0, and the other indexes were controlled under the following criteria: TG≥90 mg/dl, HDL-C≥70 mg/dl, LDL-C≤90mg/dl, then her risk of dyslipidemia 5 years after will be 7.64%, and her risk level will be in level 1(lowest level). This demonstrate that the intervention we provide is useful, can help individual reduce risk of dyslipidemia.
